# Estrogen and progesterone receptor expression levels do not differ between lobular and ductal carcinoma in patients with hormone receptor-positive tumors

**DOI:** 10.1007/s10549-017-4220-x

**Published:** 2017-04-01

**Authors:** Wilfred Truin, Rudi M. H. Roumen, Sabine Siesling, Koen K. van de Vijver, Vivianne C. G. Tjan-Heijnen, Adri C. Voogd

**Affiliations:** 1 Department of Surgery, Máxima Medical Center, Veldhoven, The Netherlands; 2 Department of Medical Oncology, GROW – School for Oncology and Developmental Biology, Maastricht University Medical Center, Maastricht, The Netherlands; 3 Department of Research, Netherlands Comprehensive Cancer Organisation, Utrecht, The Netherlands; 4 Department of Health Technology and Services Research, MIRA Institute for Biomedical Technology and Technical Medicine, University of Twente, Enschede, The Netherlands; 5 Department of Pathology, The Netherlands Cancer Institute, Amsterdam, The Netherlands; 6 Department of Epidemiology, Maastricht University Medical Center, PO Box 616, 6200 MD Maastricht, The Netherlands

**Keywords:** Invasive lobular carcinoma, Invasive ductal carcinoma, Estrogen receptor, Progesterone receptor

## Abstract

**Background:**

Differences in estrogen (ER) and progesterone (PR) expression between invasive lobular carcinoma (ILC) and invasive ductal carcinoma (IDC) could be an underlying reason for the difference in chemo-sensitivity and response to hormonal therapy between ILC and IDC. The aim of this study was to investigate the differences in ER and PR expression levels between postmenopausal patients with hormonal receptor-positive ILC and IDC.

**Methods:**

We included all ER and/or PR receptor-positive ILC and IDC, diagnosed between January 2011 and December 2013 from the population-based Netherlands Cancer Registry. A semi-quantitative classification was used to analyze differences in ER/PR expression, which consisted of three ER expression classes: 10–69, 70–89, and ≥90%. Differences in ER and PR expression levels between IDC and ILC were analyzed according to age group, tumor size, axillary nodal status, grade, and HER2 status.

**Results:**

In total, 26,339 ER and/or PR-positive breast cancers were included in the study, of which 17% were ILC and 83% IDC. In patients with IDC, 86% of the tumors showed an ER expression level of 90% or more, compared to 84% in those with ILC. In both IDC and ILC a PR expression level of 90% or more was observed in 54% of the tumors. In postmenopausal patients aged 50–69 years no significant differences could be observed in ER and PR expression levels between ILC and IDC.

**Conclusion:**

Patients with ER and PR-positive ILC and IDC have similar quantitative ER and PR expression profiles, implicating that ER/PR expression is unlikely to be a confounding factor in studies concerning chemo-sensitivity of ILC and IDC.

## Introduction

Next to invasive ductal carcinoma (IDC), invasive lobular carcinoma (ILC) is the second most common type of breast cancer, representing approximately 15% of all breast tumors. ILC is unique in its biological and clinical behavior, with a lower E-cadherin expression and a greater likelihood of being hormone receptor positive than IDC. Local control and survival is reported to be similar in patients with ILC and IDC [1, 2].

In modern treatment guidelines, no discrimination is made between ILC and IDC regarding use of systemic treatment [3], even though previous studies have shown an inferior response of estrogen (ER) and/or progesterone (PR)-positive ILC to neoadjuvant chemotherapy, compared to ER/PR-positive IDC. This difference is most pronounced when looking at the rates of pathological complete response. Reported proportions of patients with a pathological complete response range from 0 to 5% for patients with ER/PR-positive ILC, compared to 6 to 20% for those with ER/PR-positive IDC [4–6]. In contrast, significantly higher rates of pathological complete response, ranging up to almost 18%, are seen in the small portion of patients with ER/PR negative and poorly differentiated ILC, suggesting an important role for hormonal receptor status in this histological subgroup [7]. Furthermore, studies from our group showed that adjuvant chemotherapy seems to confer no additional beneficial effect to hormonal therapy in postmenopausal patients with primary non-metastatic ILC. In contrast, patients with IDC showed a relative risk reduction in mortality of about 17% with the addition of chemotherapy to hormonal treatment in the adjuvant setting [8, 9].

An important limitation in studies comparing the effect of adjuvant treatment in ILC and IDC is the lack of data concerning quantitative ER and PR levels, which could possibly be a confounding factor when interpreting these results. Quantitative data about the ER/PR expression in these patients could elucidate the question whether it is a higher ER/PR level or another intracellular signaling pathway related to histology that explains the lower chemo-sensitivity or higher response to endocrine therapy in ILC, compared to IDC. Therefore, we questioned if a difference exists in the quantitative ER and PR status of patients with ILC or IDC.

## Methods

### Patients

All female patients with ER and/or PR receptor-positive ILC or IDC diagnosed between January 2011 and December 2013 were selected from the population-based Netherlands Cancer Registry. According to Dutch guidelines, breast tumors were called ER or PR positive when the expression level was 10% or higher.

The registry records data on all patients with a new diagnosis of in situ and invasive tumors in the Netherlands. Trained registry managers prospectively collected data from medical records after notification, which are mainly obtained from the automated pathology archive (PALGA). Other sources used were the National Registry of Hospital Discharge Diagnoses and the databases of the radiotherapy departments. Data about patient, tumor, and treatment characteristics were collected from patient hospital files.

In total, 46,022 invasive breast cancers were diagnosed in the years 2011–2013. From these 46,022 breast cancers, we excluded 2271 tumors who were diagnosed together with distant metastatic disease and 3225 tumors who were not treated with surgery. From the remaining 40,526 breast cancers we excluded those for which neoadjuvant chemotherapy was used (*n* = 4789) and selected only breast cancers of either lobular or ductal histology, leaving a total 33,441 breast cancers.

### Statistical analyses

For each breast tumor, the percentage of ER and PR expression was derived from the database. The primary study endpoint of the study is the distribution of ER and PR expression levels in patients with hormone receptor-positive ILC, as compared with hormone receptor-positive IDC. To analyze differences in ER and PR expression levels between ILC and IDC, a semi-quantitative classification was used, which consisted of three ER expression classes: 10–69, 70–89 and >90%. These analyses were stratified according to age group, postoperative nodal status, postoperative tumor size, tumor grade, and HER2 status. Differences in patient characteristics between patients with IDC or ILC were calculated using the *χ* ^2^ test.

## Results

### Characteristics

ER or PR status was missing for 509 (1.5%) of the 33,441 tumors selected for the study. A positive ER status was observed in 26,118 tumors (78,1%), and a positive PR status in 21,348 tumors (63,8%). In 6645 tumors (10.9%), the ER and the PR status were both negative, and in 21,179 tumors (63.3%) both receptors were positive. In total, 26,339 ER or PR-positive breast cancers were included in the study, of which 17% were ILC and 83% IDC. Characteristics of these tumors are shown in Table 1. Age at diagnosis of patients with ILC was somewhat higher than age at diagnosis of those with IDC. Although IDCs were smaller, they were more likely to be poorly differentiated (grade 3), compared to ILCs. IDCs were more often HER2-positive, compared to ILCs.

**Table 1:** General characteristics of estrogen receptor or progesterone receptor-positive invasive lobular (ILC) or invasive ductal (IDC) breast cancers, diagnosed between 2011–2013

Characteristic	ILC (*n* = 4513)		IDC (*n* = 21,826)		*P*
	No.	%	No.	%	
Age at diagnosis (years)					
<50	675	(15)	4050	(19)	<0.0001
50–69	2512	(56)	12,348	(57)	
≥70	1326	(29)	5428	(25)	
Tumor size (cm)					
<1	691	(15)	5180	(23)	<0.0001
1–2	1710	(38)	10,438	(48)	
>2	2049	(45)	6006	(28)	
Unknown	63	(1)	202	(1)	
Axillary nodal status					
Negative	2840	(63)	14,418	(66)	0.0002
Positive	1600	(35)	7056	(32)	
Unknown	73	(2)	352	(2)	
Grade					
1	773	(17)	6364	(29)	<0.0001
2	3127	(69)	9883	(45)	
3	416	(9)	4970	(23)	
Unknown	197	(4)	609	(3)	
HER2 status					
Negative	4255	(94)	19,229	(88)	<0.0001
Positive	184	(4)	2255	(10)	
Unknown	74	(2)	342	(2)	

### ER expression in ILC and IDC

**Fig. 1:**
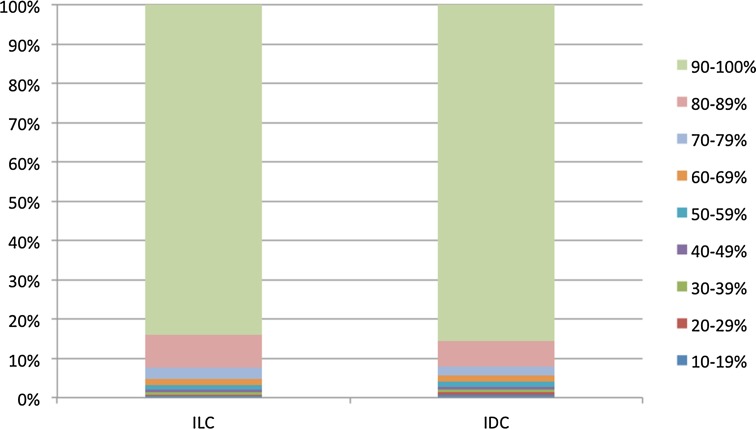
Percentage of estrogen receptor (ER) expression in ER-positive invasive lobular (ILC) or invasive ductal (IDC) breast cancer

The distribution of the ER expression level in ILC and IDC is presented in Fig. 1, and the expression levels in different subgroups is shown in Table 2. In patients aged 50–69 years, the proportion of patients with a high ER expression level (≥90%) was somewhat smaller in those with ILC than in those with IDC (84 vs. 87%, respectively). Also in patients aged 70 years or older, the group with high ER expression levels was somewhat smaller in those with ILC, compared to those with IDC (88 vs. 91%, respectively). In patients with small tumors, well-differentiated tumors, HER2-positive tumors, or a negative axillary lymph node status, the ER expression level was higher in IDC than in ILC. The ER expression level was higher in patients with ILC than in patients with IDC for those with larger tumors and poorly differentiated tumors.

**Table 2:** Percentage of estrogen receptor (ER) expression in ER-positive invasive lobular (ILC) or invasive ductal (IDC) breast cancer

Characteristic	ILC (*n* = 4498) ER expression group (%)			IDC (*n* = 21,667) ER expression group (%)			*P* value
	10–69%	70–89%	≥90%	10–69%	70–89%	≥90%	
Age at diagnosis (years)							
<50	51 (8)	113 (17)	507 (75)	443 (11)	616 (15)	2952 (74)	0.0227
50–69	121 (5)	268 (11)	2116 (84)	571 (5)	991 (8)	10,705 (87)	<.0001
≥70	39 (3)	121 (9)	1162 (88)	184 (3)	320 (6)	4887 (91)	0.0001
Tumor size (cm)							
<1	26 (4)	83 (12)	578 (84)	221 (4)	393 (8)	4537 (88)	0.0003
1–2	81 (5)	169 (10)	1456 (85)	521 (5)	880 (8)	8975 (87)	0.144
>2	99 (5)	247 (12)	1696 (83)	445 (8)	637 (11)	4858 (81)	<.0001
Axillary nodal status							
Negative	132 (5)	301 (11)	2401 (84)	709 (5)	1168 (8)	12,444 (87)	<.0001
Positive	76 (5)	195 (12)	1321 (83)	480 (7)	738 (10)	5783 (83)	0.002
Grade							
1	33 (4)	73 (9)	665 (87)	170 (3)	439 (7)	5732 (90)	0.001
2	145 (5)	338 (11)	2633 (84)	418 (4)	828 (8)	8597 (88)	<.0001
3	21 (5)	57 (14)	336 (81)	581 (12)	613 (13)	3689 (75)	0.0001
HER2 status							
Negative	183 (4)	469 (11)	3589 (85)	798 (4)	1532 (8)	16,774 (88)	<.0001
Positive	24 (13)	28 (15)	131 (72)	392 (17)	378 (17)	1457 (66)	0.199

### PR expression in ILC and IDC

**Fig. 2:**
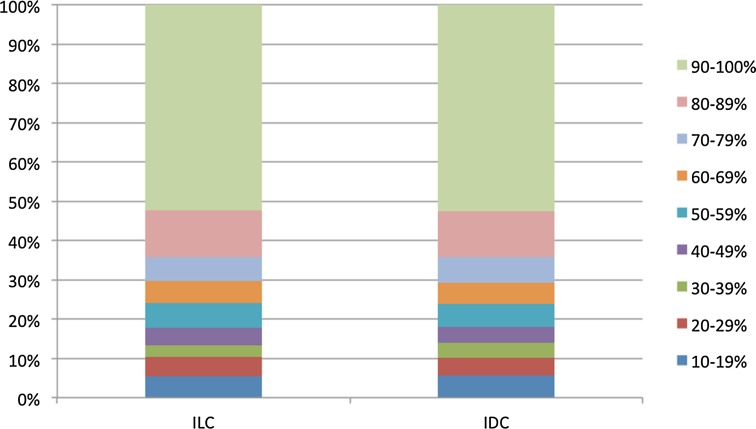
Percentage of progesterone receptor (PR) expression in PR-positive invasive lobular (ILC) or invasive ductal (IDC) breast cancer

The distribution of the PR expression level in ILC and IDC is presented in Fig. 2, and Table 3 shows the PR expression levels in different subgroups. In patients with ILC, the proportion with a high PR expression level (≥90%) was 54%, compared to a similar 54% in those with IDC. In patients aged <50 years, the proportion of patients with a high PR expression level (≥90%) was somewhat higher in those with ILC than in those with IDC (63 vs. 58%, respectively). In patients aged 70 years or older, the group with a high PR level was smaller in those with ILC, compared to those with IDC (49 vs. 54%, respectively). No remarkable differences in PR levels were observed between ILC and IDC in stratified analyses according to tumor size and axillary nodal status. The PR expression level was higher in poorly differentiated ILC, compared to poorly differentiated IDC (51 vs. 41%, respectively).

**Table 3:** Percentage of progesterone receptor (PR) expression in PR-positive invasive lobular (ILC) or invasive ductal (IDC) breast cancer

Characteristic	ILC (*n* = 3561) PR expression group (%)				IDC (*n* = 17,790) PR expression group (%)			*P* value
	10–69%	70–89%		≥90%	10–69%	70–89%	≥90%	
Age at diagnosis (years)								
<50	117 (18)	122 (19)	407 (63)		823 (23)	681 (19)	2041 (58)	0.010
50–69	615 (32)	339 (17)	989 (51)		3079 (31)	1816 (18)	4978 (50)	0.611
≥70	318 (33)	180 (19)	474 (49)		1268 (29)	761 (17)	2343 (54)	0.021
Tumor size (cm)								
<1	136 (26)	96 (18)	296 (56)		1164 (27)	740 (17)	2335 (55)	0.698
1–2	390 (28)	248 (18)	737 (54)		2371 (27)	1600 (19)	4666 (54)	0.759
>2	508 (32)	286 (18)	817 (51)		1596 (34)	896 (19)	2270 (48)	0.107
Axillary nodal status								
Negative	641 (29)	402 (18)	1171 (53)		3265 (28)	2125 (18)	6375 (54)	0.461
Positive	391 (30)	234 (18)	668 (52)		1831 (32)	1075 (19)	2836 (49)	0.329
Grade								
1	168 (28)	107 (18)	332 (55)		1295 (24)	975 (18)	3216 (59)	0.073
2	729 (30)	439 (18)	1300 (53)		2306 (28)	1496 (18)	4369 (53)	0.436
3	108 (32)	56 (17)	172 (51)		1423 (39)	713 (20)	1510 (41)	0.002
HER2 status								
Negative	984 (29)	610 (18)	1790 (53)		4390 (27)	2933 (18)	8677 (54)	0.151
Positive	47 (38)	19 (15)	57 (46)		702 (46)	288 (19)	524 (35)	0.033

## Discussion

In the present study, performed with prospectively collected data from the Netherlands Cancer Registry, statistically significant differences in ER and PR expression levels were observed between patients with ILC and IDC. However, the absolute differences were very small and are unlikely to be of clinical relevance. In ER-positive breast cancer patients, more than 80% showed an expression level of more than 90%. Especially in postmenopausal patients aged 50-69 years with ER-positive breast cancer, no significant differences could be observed in ER expression levels between ILC and IDC patients. Furthermore, the differences in PR expression levels between ILC and IDC were even smaller, compared to ER levels. We are not aware of previous studies reporting on highly detailed ER/PR expression levels of ILC and IDC in a large dataset of more than 25,000 patients.

The primary reason to perform the present study was to determine if differences in quantitative ER/PR expression between ILC and IDC could be an important limitation for the interpretation of the results of our previously published study [9]. In that study, we found that adding chemotherapy to hormonal treatment did not improve the overall survival of patients with ILC, in contrast to those with IDC, who clearly benefited from the use of chemotherapy. According to criticasters, this result could partially be explained by the better response of ILC to hormonal therapy due to higher ER expression levels, compared to patients with IDC [10]. In the present study, we proved that among patients with ER-positive breast cancer, there were no clinically relevant differences with respect to ER/PR expression levels. This conclusion suggests that it is the lobular histology itself that relates to the lower chemo-sensitivity compared to the ductal counterpart.

The discussion about the reduced chemo-sensitivity of ILC is ongoing for several years now. The added value of (neo)adjuvant chemotherapy in ER-positive ILC was already questioned in reviews by Katz et al. and Purushotham et al. [11, 12]. In several randomized neoadjuvant trials and population-based case series, patients with ILC were shown to have a significantly lower pathological complete response percentages, compared to patients with IDC [4–6]. Also in a recent, pooled analysis of nine neoadjuvant trials including 1052 patients with ILC, a low percentage of pathologic complete response was observed. However, the lower response rate did not translate into a poorer long-term outcome. Based on the lower response rates, it was recommended in this study that the use of neoadjuvant chemotherapy in patients with ILC should be restricted to the small proportion with ER-negative disease [7]. Altogether, these data contribute to the assumption that ER-positive ILC and IDC respond differently to chemotherapy.

Concerning the response of ILC and IDC to adjuvant hormonal treatment of breast cancer, a study by Rakha et al. showed that the response was better in patients with ILC and that they had a better survival as compared to matched patients with IDC [13]. A more recent study by van de Water et al. showed a similar effect of endocrine therapy regimens in IDC and ILC. On the other hand, they also reported that patients with ER-rich tumors experienced a larger benefit of upfront Exemestane, while patients with ER-poor tumors had better outcomes with sequential therapy, irrespective of histological subtype [14]. This finding emphasizes the relevance of quantification of ER expression levels in hormonal treatment strategies. In concordance with our study, van de Water et al. observed no significant differences between ILC and IDC when looking at semi-quantitative ER expression levels. Furthermore, studies investigating progesterone as a predictive marker for response to endocrine therapy show that loss of PR expression predicts relative resistance to tamoxifen, whereas maintenance of response to aromatase inhibitors can be observed, suggesting a selective role of this treatment in this subgroup [15–17].

In the current era of molecular characterization of breast cancer, most patients with ILC should be classified as luminal A, since ILC is high in ER and PR expression, often low grade and most often HER2-negative [16]. In general, luminal A type tumors do show a good responsiveness to hormonal therapy and because of this, tamoxifen and aromatase inhibitors serve as keystone therapies in ER/PR-positive ILC. This makes it difficult to prove if the good prognosis of patients with lobular breast cancer treated with endocrine therapy, and the apparent lack of an additional effect of chemotherapy, is the result of an excellent response to endocrine treatment or of a reduced chemo-sensitivity. In this light, the recent St Gallen guideline also stated that it is among these patients with the ‘luminal’ type of breast cancer, of which ILC is typical example, that uncertainty exists whether to use adjuvant chemotherapy [3].

Some limitations in this study should be considered when interpreting its results. Despite the fact that these data are derived from a large prospectively collected dataset, some missing values were observed with respect to tumor size, axillary nodal status, grade, HER2 status, and ER expression. However, the number of missing values is too small to have a real and relevant impact on the results. Moreover, information on other tumor characteristics, such as lymphovascular invasion was not available in this database.

In conclusion, our study provides strong evidence that, when looking at patients with ER and PR-positive breast cancer, ILC and IDC do not differ with respect to quantitative ER and PR expression levels. This finding provides additional proof for the lower chemo-sensitivity of ILC and the opinion that histological subtype should play an important role in the decision-making process regarding the use of chemotherapy in this patient subgroup. Future (neo)adjuvant randomized studies or analyses of existing trial data are warranted to provide further evidence on this subject.
